# Modeling and Validation of Electrostatic Sensing for UAV Targets in High-Dynamic Encounter Scenarios

**DOI:** 10.3390/s25165107

**Published:** 2025-08-17

**Authors:** Rongxiang Xia, Huifa Shi, Shaojie Ma, Feiyin Li, Yuxin Yang, He Zhang

**Affiliations:** School of Mechanical Engineering, Nanjing University of Science and Technology, Nanjing 210094, China; xrx@njust.edu.cn (R.X.); shihf@njust.edu.cn (H.S.); lifeiyinyouxiang@163.com (F.L.); 317101010026@njust.edu.cn (Y.Y.); hezhangz@njust.edu.cn (H.Z.)

**Keywords:** UAV interception, electrostatic sensor, dynamic encounter, analytical model, in-flight experiment

## Abstract

Unmanned aerial vehicles (UAVs) are increasingly used in urban management and public services, but their potential misuse poses serious risks to public safety. Electrostatic sensors offer a promising approach for UAV detection and interception by capturing their electrostatic signatures during dynamic encounters. However, the sensor output is affected by the coupling between encounter parameters and circuit characteristics, making accurate modeling challenging. This study proposes an analytical modeling method for electrically floating electrostatic sensor signals, calibrated under actual boundary conditions. The model incorporates the effects of encounter angle, miss distance, relative velocity, and equivalent input resistance-capacitance parameters, enabling efficient prediction of sensor signals under multivariable coupling. To validate the model, the electrostatic signatures during dynamic encounters were obtained using the airborne data acquisition and storage system. Results show that the predicted signals correlate well with measured data, with a correlation coefficient above 0.9. The proposed model demonstrates high computational efficiency and supports the design and optimization of electrostatic sensing systems for low-altitude UAV detection and interception.

## 1. Introduction

In recent years, unmanned aerial vehicle (UAV) technology has advanced rapidly. However, the unauthorized operation of UAVs in sensitive and restricted areas, as well as near critical infrastructure, has raised significant concerns regarding public safety and privacy [1,2,3]. Consequently, there is an urgent need for reliable methods to detect and intercept unauthorized UAVs. Due to their small size, low speed, and the use of composite materials with good electromagnetic transparency and absorption properties, small UAVs are often difficult to detect effectively using existing technologies such as radar and lidar at low altitudes. In contrast, electrostatic sensing treats ground clutter as background noise, offering inherent advantages for clutter suppression. During high-speed flight, collisions between the vehicle surface and air molecules result in electron transfer, causing electric charge accumulation on the surface [4,5,6]. Even for small UAVs, studies have shown that the amount of accumulated electric charge remains significant [7], providing a physical basis for applying electrostatic sensing techniques to UAV detection.

With the advancement of electrostatic sensing technology, electrostatic sensors have been extensively studied and applied across various domains [8,9,10], including industrial process monitoring [11,12,13], aero-engine condition diagnosis [14,15], human physiological state monitoring [16,17,18], and projectile detection [19]. Existing research on electrostatic sensing has focused on static deployments, where well-grounded sensors measure moving charged objects under simplified conditions. Typically, these studies assume a fixed encounter angle and a stationary sensor. However, in practical UAV interception scenarios, the electrically floating sensor and the UAV engage in highly dynamic encounter processes, including both parallel and non-parallel encounter conditions. In non-parallel encounters, the sensor output becomes a complex analytical function influenced by multiple variables. To improve the effectiveness of intercepting unauthorized UAVs, it is essential to develop accurate models of the sensor output signal and to conduct numerical simulations to predict electrostatic responses across different encounter scenarios.

The sensing electrodes are the core sensing components of electrostatic sensors. Analytical methods and the finite element method (FEM) are commonly used to investigate their output characteristics. Particularly when the Dirichlet boundary of the sensing electrode is irregular, deriving an analytical model is particularly challenging. FEM provides high accuracy by incorporating the actual boundary conditions, making it ideal for solving electrostatic field distributions. In industrial process measurement applications, FEM is widely used to analyze the sensing characteristics of electrostatic sensors with irregularly shaped electrodes. For example, Yan et al. applied FEM to study the response of rod-shaped electrodes in electrostatic sensors for pneumatic conveying systems, quantifying the sensor’s spatial sensitivity and bandwidth through modeling and experiments [20]. Xu et al. analyzed the influence of electrode geometry on spatial sensitivity using FEM [21], while Rahmat et al. applied this method to study annular electrodes in particle size detection within pipelines [22]. However, FEM-based models do not intuitively reveal how different parameters affect the sensor’s output signal. In addition, FEM simulations are often computationally intensive and time consuming.

In contrast, mathematical modeling allows for analytical or semi-analytical expressions of the induced charges, offering faster and more flexible analysis. Such models can incorporate a variety of influencing parameters. Murnane, for instance, developed a mirror charge model for point charges in square channels, which effectively characterized the signal types observed at the electrodes [23]. Similarly, Yan proposed a mathematical model for strip-type electrostatic sensors based on the method of image charges and the uniqueness theorem of electrostatics and further explored sensor performance in terms of spatial sensitivity and signal bandwidth [24]. Nevertheless, mathematical models often rely on idealized assumptions to simplify derivations, which can lead to accuracy limitations. Therefore, Tang et al. introduced the concept of dynamic sensitivity for measuring pipeline particles and proposed a calibration method to improve model accuracy under actual boundary conditions [25]. Comparison with simulation results demonstrated high accuracy; however, this approach is only applicable to electrostatic sensors with ideal charge preamplifiers, limiting its broader applicability.

To date, there has been no reported research specifically addressing electrically floating electrostatic sensors in high-dynamic encounter scenarios. Existing studies on electrostatic sensing systems remain limited, primarily due to the oversimplification of model parameters and the lack of experimental validation. These limitations highlight the need for more comprehensive modeling approaches and experimental investigations to support the application of electrostatic detection technology in high-dynamic encounter environments.

To address this issue, this paper proposes a practical analytical modeling method to predict the output signals of electrostatic sensors in dynamic encounter scenarios. First, a three-dimensional dynamic encounter model was developed for a flat-plate electrostatic sensor, deriving an analytical expression for the induced charge on the sensing electrode surface. The mathematical model was further calibrated using high-accuracy FEM simulations. Subsequently, based on the calibrated induced charge function, an output response model was developed, incorporating encounter angle, miss distance, relative velocity, and equivalent input resistance–capacitance parameters to simulate sensor responses under various encounter scenarios. Finally, a high-dynamic encounter experimental method was proposed. A modified miniature rocket equipped with an electrostatic sensor featuring data acquisition and storage was used to record in-flight electrostatic signals. A comparative analysis between experimental data and simulation results was conducted to assess the accuracy and applicability of the proposed model. This work also aims to evaluate the feasibility of electrostatic sensing in low-altitude dynamic encounter scenarios, providing practical guidance for the design of UAV detection and interception sensing systems.

## 2. Modeling of Electrostatic Sensing in Dynamic Encounter Scenarios

### 2.1. Principle of the Flying-Net Interception Device Based on Electrostatic Sensor

**Figure 1:**
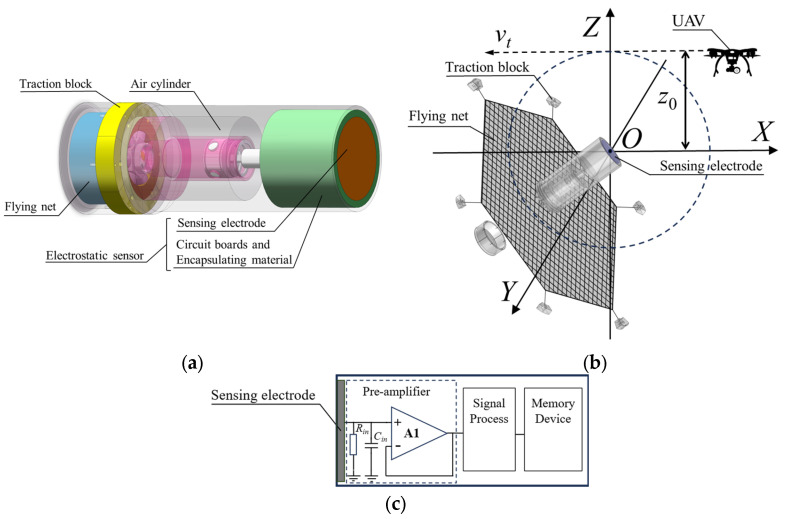
Structure and principle of the flying-net interception device based on electrostatic sensor: (**a**) diagram of the flying-net interception device; (**b**) schematic illustration of the dynamic encounter; (**c**) schematic diagram of the electrostatic sensor.

The electrostatic sensor investigated in this study adopts a planar electrode configuration, powered by a battery, and operates in an electrically floating state. It is powered by a battery and operates in an electrically floating state. The sensor is installed at the front end of a flying-net interception device. Simplified schematics of the device structure, operating principle, and electrostatic sensor design are shown in Figure 1a, Figure 1b, and Figure 1c, respectively.

As shown in Figure 1a, the flying-net interception device is designed for launch from aerial or ground-based unmanned platforms. It consists of an electrostatic sensor, a high-pressure gas cylinder, a piston release mechanism, traction blocks, and a deployable capture net. The electrostatic sensor itself comprises the sensing electrode, a metallic shell, encapsulated dielectric materials for mechanical buffering and insulation, and a signal conditioning circuit. In Figure 1b, *v_t_* denotes the relative encounter velocity, and *z*_0_ represents the miss distance, with the geometric center of the sensing electrode taken as the origin of the Cartesian coordinate system. When an electrostatically charged aerial object, such as an unauthorized UAV, enters the sensing region, the charges on its surface induce corresponding charges on the sensing electrode. The induced charges are processed by the conditioning circuit shown in Figure 1c, which includes charge-to-voltage conversion, digital signal processing, and data storage. When the processed signal indicates that the target has entered the effective capture range, the piston mechanism is triggered, launching the traction blocks via high-pressure gas to deploy the capture net.

### 2.2. Dynamic Encounter Model of Electrostatic Sensor

#### 2.2.1. Mathematical Model

To derive an analytical expression for the variation in induced charge on the sensing electrode, several simplifications are introduced. The electrostatically charged aerial object (i.e., unauthorized UAV) is modeled as an ideal point charge *Q*, neglecting its geometric size. The distortion of the electrostatic field caused by the sensor housing is neglected. The sensing electrode is idealized as an infinite conducting plane, and its thickness, dielectric layer, and guard ring effects are excluded.

**Figure 2:**
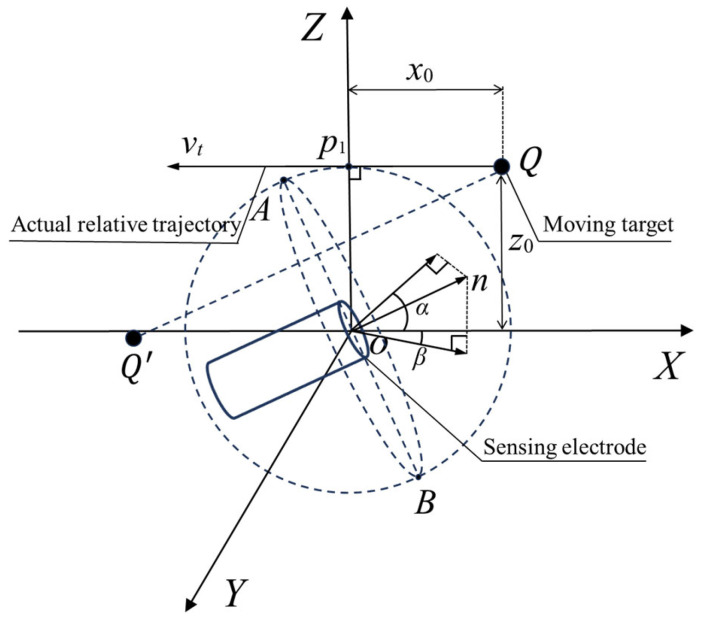
Geometric model of dynamic encounter in 3D coordinates.

As shown in Figure 2, the center of the circular electrode is defined as the origin of a right-handed Cartesian coordinate system. The target is modeled as a point charge with magnitude *q*. The miss distance is denoted as *z*_0_, and the relative encounter velocity *v_t_* is aligned with the negative X-axis. The unit normal vector $\vec{n}$ denotes the direction of the sensor’s axis, which is perpendicular to the electrode plane. The sensing electrode center is located at the origin, with the electrode radius denoted as *r*_0_.

The image charge *Q’* is placed symmetrically with respect to the electrode plane and has the same magnitude but opposite polarity as the real charge *Q*. To account for angular misalignments during the encounter, the angle of attack *α* is defined as the angle between the projection of $\vec{n}$ onto the *XOZ*-plane and the positive *X*-axis, while the sideslip angle *β* is the angle between the projection of $\vec{n}$ onto the *XOY*-plane and the *X*-axis.

The electrostatic potential at a point *P* (*x*, *y*, *z*) due to charge *Q* (*x*_0_, *y*_0_, *z*_0_) and its image charge *Q*′ (*x*_1_, *y*_1_, *z*_1_) is given by the following:

(1) $$\begin{array}{cc} \varphi \left(P\right) & =\frac{q}{4\pi \varepsilon_{0}\sqrt{{\left(x-x_{0}\right)}^{2}+{\left(y-y_{0}\right)}^{2}+{\left(z-z_{0}\right)}^{2}}}\\ & -\frac{q}{4\pi \varepsilon_{0}\sqrt{{\left(x-x_{1}\right)}^{2}+{\left(y-y_{1}\right)}^{2}+{\left(z-z_{1}\right)}^{2}}}+C \end{array}$$

where *ε* is the permittivity of the surrounding medium, and *C* is the potential constant for the ungrounded conductor plane. The coordinates of the image charge *Q*′ are determined by the following:

(2) $$\left\{\begin{array}{l} \left[\begin{array}{c} x_{1}\\ y_{1}\\ z_{1} \end{array}\right]=\left[\begin{array}{c} x_{0}\\ y_{0}\\ z_{0} \end{array}\right]-2\cdot d\cdot \hat{n}\\ d=x_{0}\cos\alpha \cos\beta +y_{0}\cos\alpha \sin\beta +z_{0}\sin\alpha \\ \vec{n}=\left[\begin{array}{c} \cos\alpha \cos\beta \\ \cos\alpha \sin\beta \\ \sin\alpha \end{array}\right] \end{array}\right.$$

where *d* is the perpendicular distance from *Q* to the electrode plane. The normal component *E_n_* of the electric field on the electrode surface is given by the following:

(3) $$\left\{\begin{array}{c} E_{n}=\left(E_{x},E_{y},E_{z}\right)\cdot \left(\cos\alpha \cos\beta ,\cos\alpha \sin\beta ,\sin\alpha \right)\\ =E_{x}\cos\alpha \cos\beta +E_{y}\cos\alpha \sin\beta +E_{z}\sin\alpha \\ E_{x}=-\frac{\partial \varphi \left(P\right)}{\partial x}\\ E_{y}=-\frac{\partial \varphi \left(P\right)}{\partial y}\\ E_{z}=-\frac{\partial \varphi \left(P\right)}{\partial z} \end{array}\right.$$

Thus, the total induced charge *q*_1_ on the electrode surface is as follows:

(4) $$\begin{array}{c} q_{1}=\int_{A}\sigma dS=\int_{A}\varepsilon_{0}E_{n}dS\\ =\frac{q}{4\pi }\int_{A}\left(\frac{-\left(x-x_{0}\right)\cos\alpha \cos\beta -\left(y-y_{0}\right)\cos\alpha \sin\beta -\left(z-z_{0}\right)\sin\alpha }{{\left({\left(x-x_{0}\right)}^{2}+{\left(y-y_{0}\right)}^{2}+{\left(z-z_{0}\right)}^{2}\right)}^{3/2}}\right.\\ \left.+\frac{\left(x-x_{1}\right)\cos\alpha \cos\beta +\left(y-y_{1}\right)\cos\alpha \sin\beta +\left(z-z_{1}\right)\sin\alpha }{{\left({\left(x-x_{1}\right)}^{2}+{\left(y-y_{1}\right)}^{2}+{\left(z-z_{1}\right)}^{2}\right)}^{3/2}}\right)dS \end{array}$$

For a circular electrode of radius *r*_0_, the surface can be parameterized using orthonormal vectors $\vec{u}$, $\vec{v}$ lying in the electrode plane:

(5) $$\left\{\begin{array}{l} r\left(\theta \right)=r\left(\vec{u}\cos\theta +\vec{v}\sin\theta \right)\\ \vec{u}=\left[\begin{array}{c} -\sin\beta \\ \cos\beta \\ 0 \end{array}\right],\vec{u}=\left[\begin{array}{c} \sin\alpha \cos\beta \\ \sin\alpha \sin\beta \\ -\cos\alpha \end{array}\right]\\ \theta \in \left[0,2\pi \right]\\ \rho \in \left[0,r_{0}\right] \end{array}\right.$$

Using polar coordinates, the final expression for the induced charge under both parallel and non-parallel encounter conditions is as follows:

(6) $$\begin{array}{l} q_{1}=\int_{0}^{2\pi }\int_{0}^{r_{0}}\sigma \left(r,\theta \right)rdrd\theta \\ =\frac{q}{4\pi }\int_{0}^{2\pi }\int_{0}^{r_{0}}\left(\frac{\begin{array}{l} -\left(\rho \left(-cos\theta sin\beta +sin\theta sin\alpha cos\beta \right)-x_{0}\right)\cos\alpha \cos\beta \\ -\left(\rho \left(cos\theta cos\beta +sin\theta sin\alpha sin\beta \right)-y_{0}\right)\cos\alpha \sin\beta +\left(\rho \left(sin\theta cos\alpha \right)+z_{0}\right)\sin\alpha \end{array}}{{\left(\begin{array}{l} {\left(\rho \left(-cos\theta sin\beta +sin\theta sin\alpha cos\beta \right)-x_{0}\right)}^{2}\\ +{\left(\rho \left(cos\theta cos\beta +sin\theta sin\alpha sin\beta \right)-y_{0}\right)}^{2}+{\left(\rho \left(sin\theta cos\alpha \right)+z_{0}\right)}^{2} \end{array}\right)}^{3/2}}\right.\\ \left.+\frac{\begin{array}{l} \left(\rho \left(-cos\theta sin\beta +sin\theta sin\alpha cos\beta \right)-x_{1}\right)\cos\alpha \cos\beta \\ +\left(\rho \left(cos\theta cos\beta +sin\theta sin\alpha sin\beta \right)-y_{1}\right)\cos\alpha \sin\beta -\left(\rho \left(sin\theta cos\alpha \right)+z_{1}\right)\sin\alpha \end{array}}{{\left(\begin{array}{l} {\left(\rho \left(-cos\theta sin\beta +sin\theta sin\alpha cos\beta \right)-x_{1}\right)}^{2}\\ +{\left(\rho \left(cos\theta cos\beta +sin\theta sin\alpha sin\beta \right)-y_{1}\right)}^{2}+{\left(\rho \left(sin\theta cos\alpha \right)+z_{1}\right)}^{2} \end{array}\right)}^{3/2}}\right)rdrd\theta \end{array}$$

Spatial sensitivity is a key performance metric of an electrostatic sensor, representing its ability to respond to external charges at different locations. Accurate modeling and optimization of spatial sensitivity are critical for both sensor design and operational performance. The spatial sensitivity *S* at a point is defined as the absolute ratio of the induced charge *q*_1_ on the sensing electrode produced by an external point charge *q* [26]. Equation (6) provides an analytical expression for the induced charge on the electrode, based on the assumption of an infinite conducting plane. Therefore, the analytical spatial sensitivity of the electrostatic sensor can be expressed as follows:

(7) $$S\left(x_{0}{,y}_{0}{,z}_{0}\right)=\left|\frac{q_{1}\left(x_{0}{,y}_{0}{,z}_{0}\right)}{q}\right|$$

However, in practical scenarios, the electrostatic sensor is mounted on an aerial platform, with its metallic shell sharing a common ground with the interception device body, which is electrically floating relative to the Earth. Therefore, the actual electrostatic boundary conditions deviate from the idealized assumptions in the model. To address this discrepancy, finite element modeling (FEM) is used to calibrate the analytical results and obtain a more accurate response model for dynamic encounter scenarios.

#### 2.2.2. Model Calibration Based on Actual Boundary Conditions

In the previous section, we established a simplified analytical dynamic encounter model. Due to the complex three-dimensional electric field distribution surrounding the sensor and its mounting platform, deriving a closed-form analytical solution is generally difficult. Therefore, in this study, the induced charge on the sensing electrode was calibrated using FEM.

Given that electrostatic equilibrium is reached almost instantaneously (on the order of 10^−19^ s), the interaction between the electrostatic sensor and a charged aerial object in space can be effectively modeled as an electrostatic problem [25]. The system is uniquely defined by the Poisson equation with Dirichlet boundary conditions:

(8) $$\nabla^{2}\varphi =-\frac{\rho }{\varepsilon }$$

(9) $$\left\{\begin{array}{l} \varphi_{\Gamma_{1}}={\mathrm{const}}_{1}\\ \varphi_{\Gamma_{2}}={\mathrm{const}}_{2}\\ E_{\infty }=0 \end{array}\right.$$

Here, $\Gamma_{1}$ denotes the surface of the sensing electrode, $\Gamma_{2}$ denotes the metallic casing of the interception device, ρ is the charge density, φ is the electrostatic potential distribution, $E_{\infty }$ is the electric field intensity at infinity, and ε is the dielectric constant. The constant terms const_1_ and const_2_ represent the equipotential surfaces of the sensing electrode and the metallic housing.

The spatial sensitivity defined in Equation (7) is based on a simplified boundary condition that idealizes the electrode plane as an infinite conducting surface. This approach neglects distortion of the spatial electric field caused by the metallic housing and approximates the edge effects of the actual conductor. However, during dynamic encounters, the electrostatic sensor is mounted on a floating-potential carrier with complex geometric features. To improve the accuracy of the analytical model under the actual boundary conditions, correction terms *l* and $k\left(x_{0},y_{0},z_{0}\right)$ are introduced into the spatial sensitivity function, yielding a calibrated spatial sensitivity model expressed as follows:

(10) $$S_{c}\left(x_{0},y_{0},z_{0}\right)=k\left(x_{0},y_{0},z_{0}\right)S\left(x_{0},y_{0},z_{0},l\right)$$

The correction process involves the following steps:

First, a grid of spatial nodes is established across the computational cross-section to fully encompass the domain of interest. At each node, the theoretical spatial sensitivity $S\left(x_{0},y_{0},z_{0}\right)$ is computed using the simplified analytical model. Next, a finite element model is built, incorporating the actual boundary conditions (floating potential for the electrode and the platform housing). Then, the simulated spatial sensitivity values at each grid node are obtained from the FEM results. After that, the zero spatial sensitivity plane (where the induced charge goes to zero) is identified in the FEM model. In the analytical method, this plane corresponds to either the sensing electrode itself or to an idealized image plane at infinity, consistent with the assumptions of the method of image charges. Subsequently, a displacement correction term *l* is introduced to ensure consistency between the geometrical configurations of the analytical and simulation models. This step guarantees that both spatial sensitivity datasets are evaluated within the same reference frame. Finally, the deviation between the theoretical and simulated sensitivities is computed at each grid point, and the correction term $k\left(x_{0},y_{0},z_{0}\right)$ is obtained through linear or nonlinear regression fitting.

**Figure 3:**
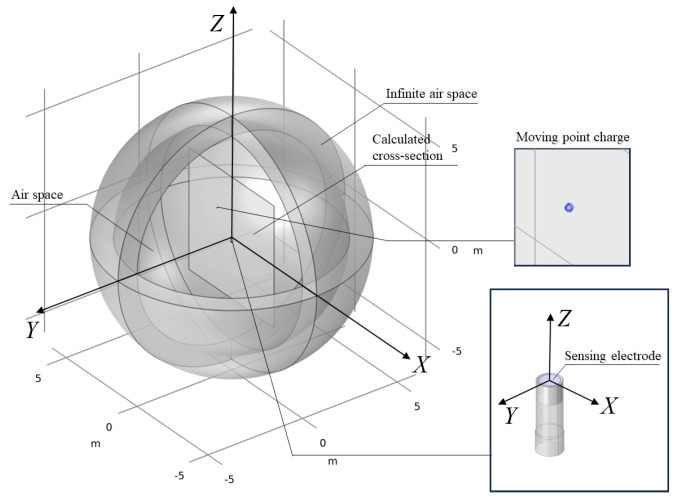
The FEM simulation model under the actual boundary condition.

As shown in Figure 3, the FEM model includes both the sensor and the interception device. Since the housing of the electrostatic sensor is mechanically integrated with the interceptor body via a threaded connection system, the simulation model is divided into three main components: the sensing electrode, the epoxy encapsulation material, and the metallic housing. The sensing electrode has a diameter of 60 mm, while the metallic housing has a diameter of 100 mm and a height of 355 mm (see Table 1). Both the sensing electrode surface and the housing are set to floating potential, with the housing treated as an equipotential body. The surrounding dielectric constant is set to 1 (air), while the encapsulation material of the sensor circuit has a relative permittivity of 4.5 (the sensor circuit board was encapsulated with epoxy resin, forming a monolithic integrated structure). A spherical body with a unit charge (1 C) is used to represent a point charge, so that the absolute value of the total induced charge directly corresponds to the spatial sensitivity.

**Figure 4:**
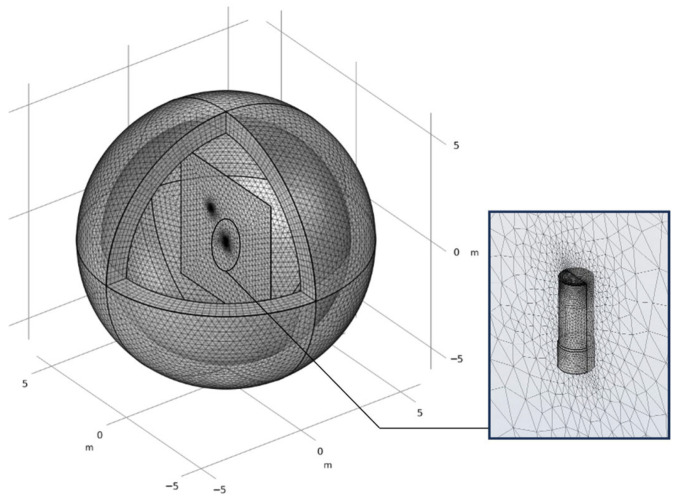
Schematic diagram of the finite element mesh used in the simulation.

To improve numerical accuracy, mesh refinement is applied near the point charge and electrode surface, where the electric field varies rapidly. The model is discretized using a free tetrahedral mesh with approximately 140,000 elements (see Figure 4).

**Figure 5:**
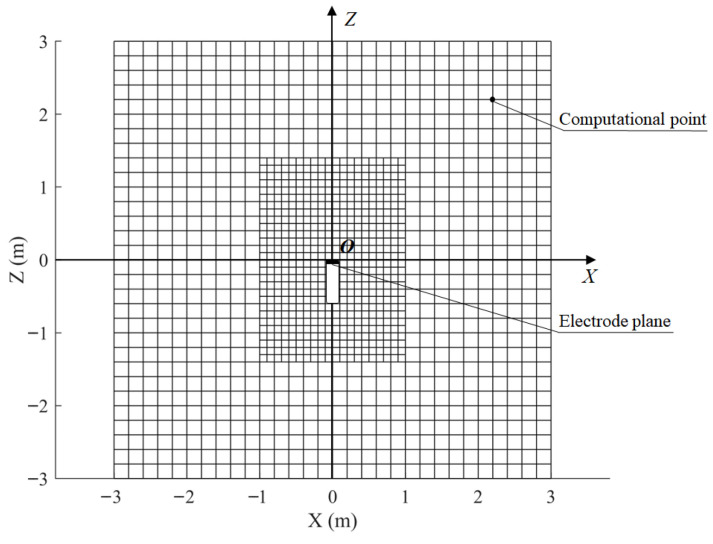
The grid layout in a computational plane.

Considering the axial symmetry of the sensor, the three-dimensional spatial sensitivity distribution can be represented using a two-dimensional cross-section. A 6 m × 6 m computational cross-section is defined in the *XOZ*-plane, consisting of 1320 points with higher mesh density near the sensor region. The mesh layout is illustrated in Figure 5. Benefiting from the symmetry of the model, only the points on one side of the plane symmetric about the *Z*-axis need to be computed, thereby reducing the number of effective computation points to 660.

**Figure 6:**
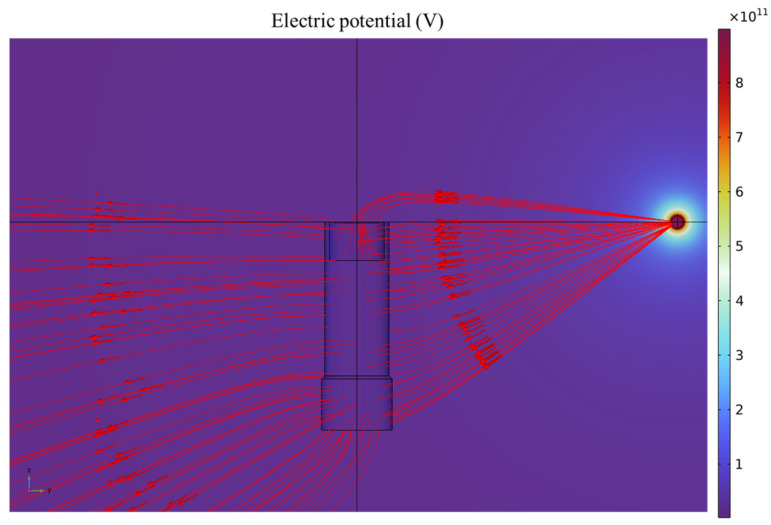
Electric field distribution around the sensor.

When a charged object is positioned at coordinates (0.5, 0, 0), its electric field distribution is shown in Figure 6. At this position, the charged object and the sensing electrode lie in the same plane. The red streamlines with arrows represent the electric field lines, with the arrows indicating the field direction. These field lines terminate at the sensing electrode, the metallic enclosure, the ground, or at infinity. To present the spatial distribution of the electric field between the charged object, sensing electrode, and metallic enclosure more clearly and intuitively, Figure 6 only displays the electric field lines located on the surfaces of the sensing electrode and metallic housing, as well as those between them, while the lines extending from the charged object to infinity are omitted. The electric field lines between the sensing electrode and the metallic housing indicate capacitive coupling between the two. As the charged object moves, the potentials on both the sensing electrode and the metallic housing vary accordingly. Using finite element simulations, the coupling capacitance between the sensing electrode and the housing, as well as their respective potentials, can be obtained. These values are then used to calculate the induced charge measured by the sensor and to determine its spatial sensitivity (see Appendix A for the derivation and calculation procedure).

**Figure 7:**
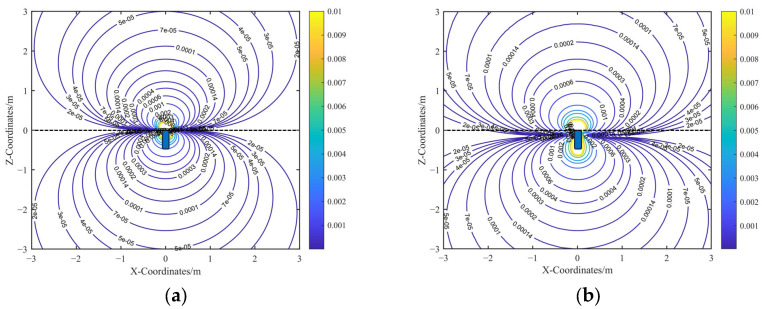
Spatial sensitivity contour diagram before calibration: (**a**) Contour diagram of the uncalibrated analytical spatial sensitivity. (**b**) Contour diagram of the FEM-simulated spatial sensitivity.

The spatial sensitivity contour map over the computational cross-section is shown in Figure 7. In contrast to electrostatic sensors with grounded housing boundaries, electrically floating electrostatic sensors exhibit both forward and backward spatial sensitivity. Moreover, in the far-field region, the spatial sensitivity exhibits symmetry with respect to the zero spatial sensitivity plane. Specifically, in the far-field region, any pair of points symmetric about this plane induces equal-magnitude but opposite-polarity charges on the sensing electrode.

A comparison between the analytical and FEM simulation results shows that the analytical spatial sensitivity gradient is symmetric about the Z = 0 plane, which corresponds to the electrode surface in the analytical model. In contrast, the FEM simulation exhibits a slight shift in the symmetry plane to Z = −0.15 m in the far field due to boundary effects. Nevertheless, both the analytical and simulated sensitivity gradients maintain symmetric distributions in the far field, centered around their respective zero-sensitivity planes.

In the far-field region where the miss distance *z*_0_ is much greater than the electrode radius *r*_0_, the analytical model for the induced charge can be further simplified:

(11) $$q_{1}=\int_{0}^{2\pi }\int_{0}^{r_{0}}\sigma \left(r,\theta \right)rdrd\theta =\frac{{\mathrm{qr}}_{0}^{2}\left(cos\alpha cos\beta \cdot x_{0}+cos\alpha sin\beta \cdot y_{0}+sin\alpha \cdot z_{0}\right)}{2{\left(x_{0}^{2}+y_{0}^{2}+z_{0}^{2}\right)}^{3/2}}$$

A displacement correction term *l* is introduced to the model, yielding the following:

(12) $$\left\{\begin{array}{l} \left[\begin{array}{c} x_{1c}\\ y_{1c}\\ z_{1c} \end{array}\right]=\left[\begin{array}{c} x_{0}\\ y_{0}\\ z_{0} \end{array}\right]-2\cdot d_{c}\cdot \vec{n}\\ d_{c}=x_{0}\cos\alpha \cos\beta +y_{0}\cos\alpha \sin\beta +z_{0}\sin\alpha -l\\ \vec{n}=\left[\begin{array}{c} \cos\alpha \cos\beta \\ \cos\alpha \sin\beta \\ \sin\alpha \end{array}\right] \end{array}\right.$$

**Figure 8:**
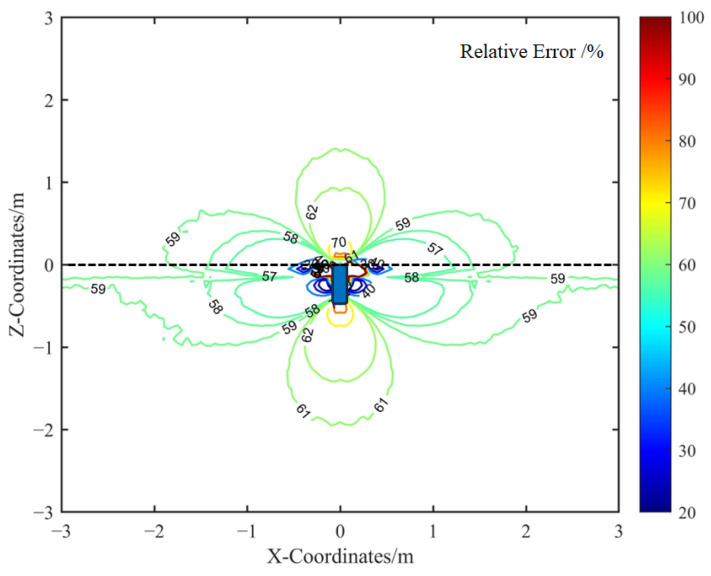
Relative error between the analytical model with the displacement correction term *l* and the FEM model.

As shown in Figure 8, introducing the displacement correction term *l* establishes a clear linear relationship between the analytical model and the FEM results in the far-field region. To preserve the simplicity of the corrected analytical expression, a proportional correction approach is adopted, introducing a correction coefficient *k*, which serves as a proportionality constant calibrated using FEM results. The final form of the corrected analytical expression for the induced charge on the sensing electrode is given by the following:

(13) $$q_{1c}=\frac{{\mathrm{kqr}}_{0}^{2}\left(cos\alpha cos\beta \cdot x_{0}+cos\alpha sin\beta \cdot y_{0}+sin\alpha \cdot z_{0}+l\right)}{2{\left(x_{0}^{2}+y_{0}^{2}+z_{0}^{2}\right)}^{3/2}}$$

**Figure 9:**
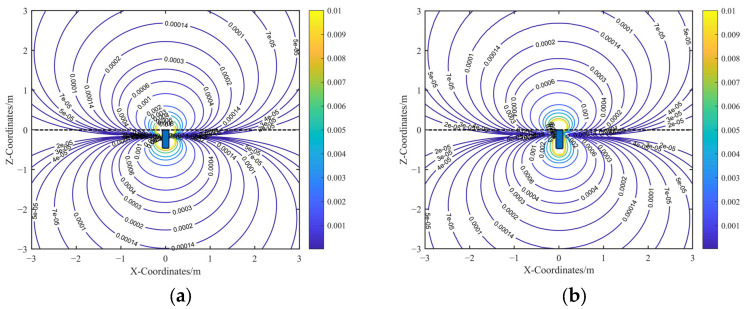
Spatial sensitivity contour diagram after calibration: (**a**) Contour diagram of the calibrated analytical spatial sensitivity. (**b**) Contour diagram of the FEM-simulated spatial sensitivity.

The contour plots of the corrected analytical spatial sensitivity and the FEM-simulated spatial sensitivity are presented in Figure 9. Comparing Figure 9a,b shows that the calibrated analytical sensitivity closely matches the simulated spatial sensitivity in the far-field region.

**Figure 10:**
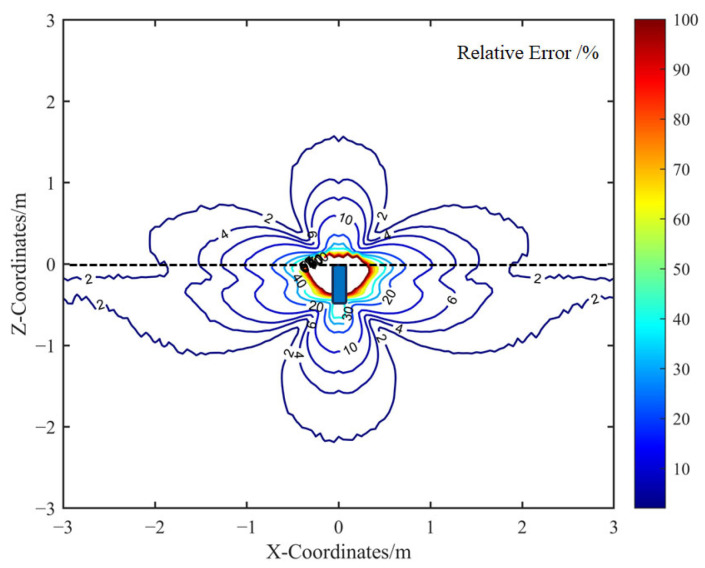
Relative error between the calibrated analytical model and the FEM model.

Furthermore, Figure 10 illustrates the spatial distribution of the relative error between the calibrated analytical model and the FEM simulation. The comparison indicates that the error is more pronounced near the metallic housing, primarily because the analytical model is formulated for far-field conditions. To maintain the simplicity of the analytical expression, corrections for near-field effects were intentionally omitted.

In regions where the miss distance between the sensor and the target exceeds 1 m, the relative error of the calibrated analytical spatial sensitivity remains below 8%. This level of accuracy is sufficient for the practical analysis of electrostatic sensor responses in dynamic encounter scenarios.

## 3. Results and Discussion

### 3.1. Output Signal Analysis of the Electrostatic Sensor

The input current *i*(*t*) to the preamplifier during the dynamic encounter process is expressed as follows:

(14) $$i\left(t\right)=\frac{dq_{1c}\left(t\right)}{dt}=\frac{kqr_{0}^{2}}{2}\cdot \frac{\left(\begin{array}{l} v_{t}(v_{t}^{2}t^{2}+y_{0}^{2}+z_{0}^{2})cos\alpha cos\beta \\ -3v_{t}^{2}t(v_{t}tcos\alpha cos\beta +y_{0}sin\beta cos\alpha +z_{0}sin\alpha +l) \end{array}\right)}{{\left(v_{t}^{2}t^{2}+y_{0}^{2}+z_{0}^{2}\right)}^{5/2}}$$

As illustrated in the equivalent circuit diagram of the preamplifier in Figure 1c, *R_in_* and *C_in_* represent the equivalent input resistance and capacitance, respectively, with the associated time constant defined as $\tau =R_{in}C_{in}$. The output voltage signal *U*(*t*) of the preamplifier is described by the following expression:

(15) $$\begin{array}{cc} U(t) & =\frac{e^{-\frac{t}{\tau }}}{C_{in}}\int_{t_{0}}^{t}i\left(x\right)e^{\frac{x}{\tau }}dx\\ & =\frac{kqr_{0}^{2}e^{-\frac{t}{\tau }}}{2C_{in}}\int_{t_{0}}^{t}\frac{\left(\begin{array}{c} v(v^{2}x^{2}+y_{0}^{2}+z_{0}^{2})cos\alpha cos\beta \\ -3v^{2}x(vxcos\alpha cos\beta +y_{0}sin\beta cos\alpha +z_{0}sin\alpha +l) \end{array}\right)\cdot e^{\frac{x}{\tau }}}{{\left(v^{2}x^{2}+y_{0}^{2}+z_{0}^{2}\right)}^{5/2}}dx \end{array}$$

Thus, the induced charge *q*_1_*c* on the sensing electrode is converted into the output voltage *U*(*t*) through two sequential transfer functions. The first transfer function *f*_1_ represents the differentiation stage, capturing the relationship between the induced charge and the resulting current. The second transfer function *f*_2_ models the parallel connection of *R_in_* and *C_in_*, converting the current signal into a voltage signal.

The overall transfer function of the preamplifier system is the cascade of *f*_1_ and *f*_2_, which yields the following:

(16) $$H\left(S\right)=f_{1}(s)\cdot f_{2}(s)=\frac{sR_{in}}{1+sR_{in}C_{in}}$$

**Figure 11:**
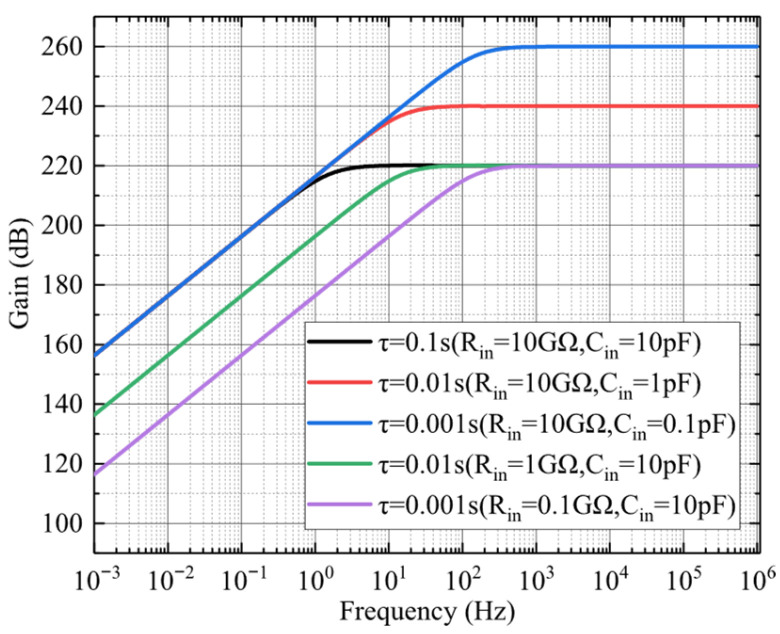
Amplitude–frequency response of the preamplifier in the electrostatic sensor.

The overall transfer function of the electrostatic sensor’s preamplifier circuit exhibits the characteristics of a typical first-order high-pass filter, with a cutoff frequency defined as $f_{c}=1/\left(2\pi R_{in}C_{in}\right)$. The frequency response of the transfer function is presented in Figure 11. As shown, the passband gain is primarily determined by the equivalent input capacitance *C_in_*, with smaller values of *C_in_* resulting in higher gain.

To investigate the effects of equivalent input RC parameters (*R_in_* and *C_in_*) on the output signals, the following conditions were assumed: *α* = 0°, *β* = 0°, *r*_0_ = 0.06 m, z_0_ = 1.5 m, *v_t_* = 500 m/s, and *q* = 1 × 10^−9^ C.

**Figure 12:**
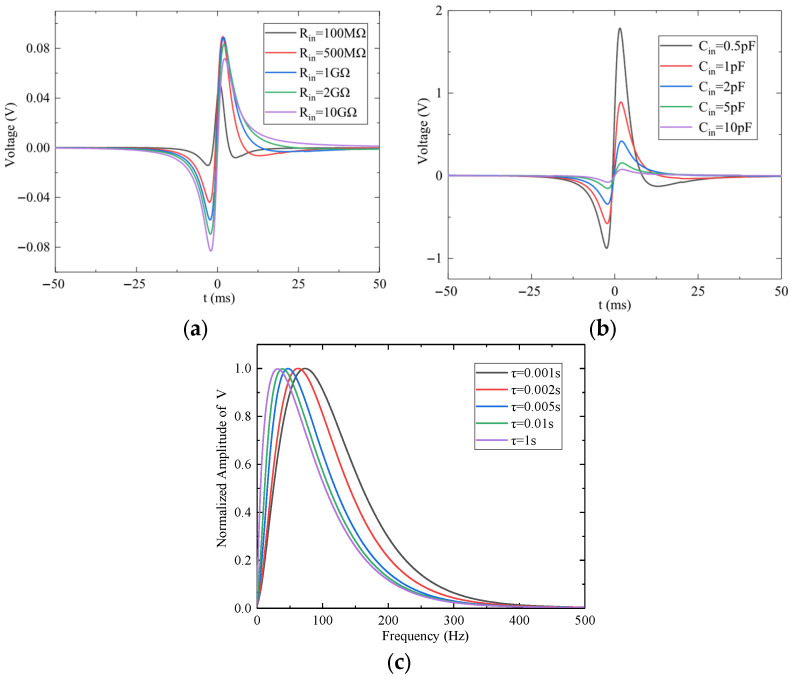
The effect of equivalent input RC on the output signal of the preamplifier: (**a**) Time-domain response for different input resistance values. (**b**) Time-domain response for different input capacitance values. (**c**) Frequency response for different time constants *τ*.

As shown in Figure 12a, when *C_in_* = 10 pF, increasing *R_in_* reduces the cutoff frequency, thereby broadening the passband and making the output waveform more closely match the induced charge signal. Figure 12b demonstrates that, with *R_in_* = 10 GΩ, reducing *C_in_* increases the gain and enhances the signal amplitude. The normalized spectra of the output signals under different time constants *τ* are presented in Figure 12c. As *τ* decreases, the cutoff frequency increases, resulting in higher signal frequencies.

Overall, when the bandwidth of the induced charge signal overlaps with the preamplifier’s stopband, the signal current primarily flows through *R_in_*, causing the circuit to operate as a current amplifier. Conversely, when the signal bandwidth falls within the passband, the path is dominated by *C_in_*, and the circuit functions as a potential amplifier. Proper tuning of *R_in_* and *C_in_* can therefore reduce output signal distortion introduced by the circuit’s filtering characteristics.

To analyze the sensor’s response under non-parallel dynamic encounter conditions, the effects of variations in angle of attack (*α*) and sideslip angle (*β*) on the output signal were examined under fixed parameters: *r*_0_ = 0.06 m, *z*_0_ = 1.5 m, *v_t_* = 500 m/s, *q* = 1 × 10^−9^ C, *R_in_* = 10 GΩ, and *C_in_* = 10 pF.

**Figure 13:**
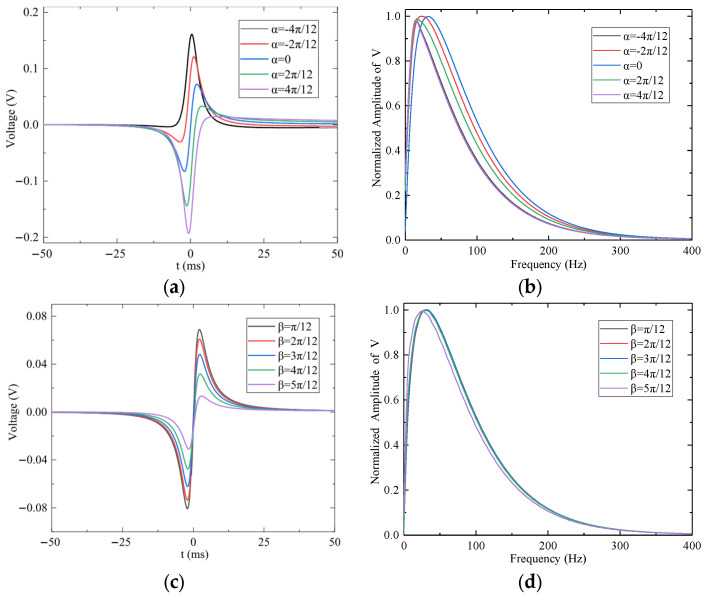
The effect of attack angle α and sideslip angle *β* on the output signal of the preamplifier: (**a**) Time-domain response for different *α* values. (**b**) Frequency response for different *α* values. (**c**) Time-domain response for different β values. (**d**) Frequency response for different β values.

The effect of the angle of attack (*α*) was analyzed with sideslip angle β = 0°. Figure 13a shows that increasing *α* enhances the coupling between the sensing electrode and the point charge, resulting in an approximately linear increase in the amplitude of the first signal extremum (negative peak). Figure 13b presents the normalized frequency spectra, indicating that the signal’s dominant frequency decreases with increasing |*α*|. As illustrated in Figure 13c,d, increasing the sideslip angle *β* from 15° to 75° causes an approximately linear decrease in the amplitude of the first extremum. Frequency-domain analysis indicates that the dominant frequency shifts are minimal (<5 Hz), suggesting that β primarily affects signal amplitude rather than its spectral distribution.

**Figure 14:**
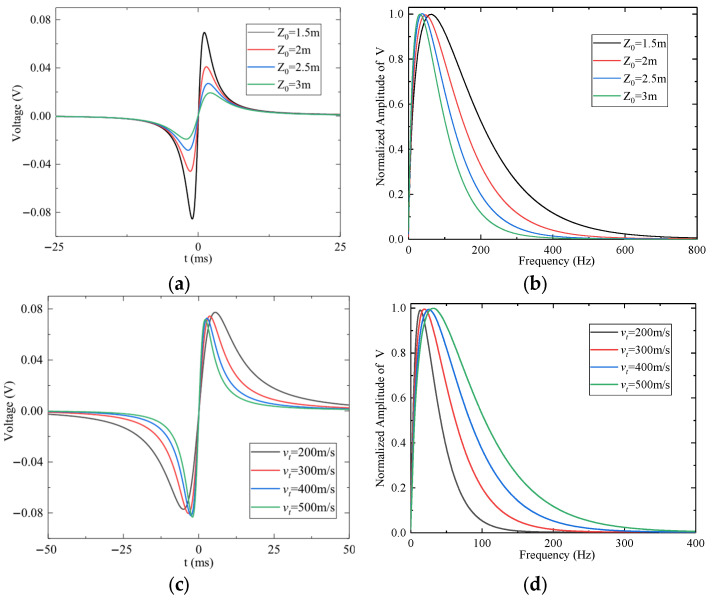
The effect of miss distance *z*_0_ and encounter velocity *v_t_* on the output signal of the preamplifier: (**a**) Time-domain response for different *z*_0_ values. (**b**) Frequency response for different *z*_0_ values. (**c**) Time-domain response for different *v_t_* values. (**d**) Frequency response for different *v_t_* values.

The effect of miss distance (*z*_0_) on the sensor output is shown in Figure 14a,b, with *α* = 0° and β = 0°. As *z*_0_ increases, the coupling between the charge and the electrode weakens, and the amplitude of the first signal extremum decreases approximately in proportion to 1/*z*_0_^2^. The normalized frequency spectrum in Figure 14b confirms that the dominant frequency decreases as *z*_0_ increases. Figure 14c,d demonstrate the influence of encounter velocity (*v_t_*) on the output signal. As shown, increasing *v_t_* raises the dominant frequency of the signal while having minimal impact on its amplitude.

### 3.2. In-Flight Measurement Experiment of the Electrostatic Sensor

**Figure 15:**
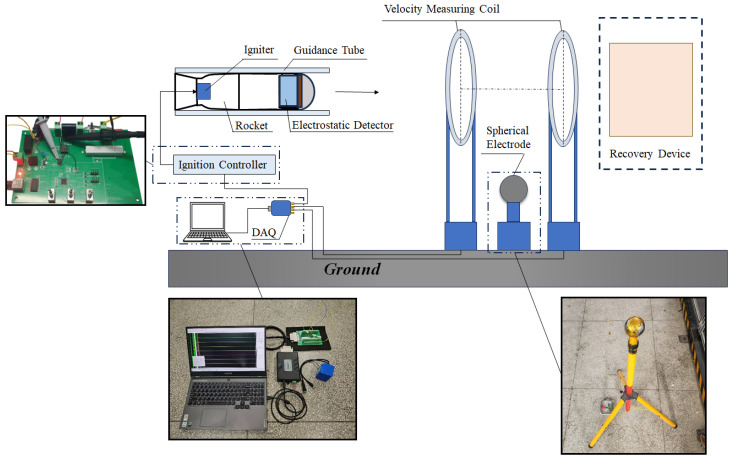
Schematic diagram of the full-flight measurement experiment plan.

To validate the proposed model and obtain the in-flight signal response of the electrostatic sensor during a dynamic encounter, an experiment was conducted as shown in Figure 15. A modified miniature rocket served as the sensor carrier, while a spherical metal electrode with an applied electric potential simulated the electrostatic target. The spherical electrode was precisely positioned at the center of a pair of induction coils, which were used to determine the timing of the sensor’s approach to and departure from the simulated electrostatic target. Additionally, a ground-based data acquisition system simultaneously records signals from both the induction coils and the ignition control system via coaxial cables.

To ensure accurate synchronization of data acquisition and storage timing between the electrostatic sensor’s onboard system and the ground-based data acquisition system, the ignition signal was utilized to trigger the data recording operation. Following this triggering event, the onboard system initiates continuous storage of acquired signal data into its dedicated internal memory.

To simulate an electrostatically charged aerial object, a controllable electric potential *U_s_* was applied to the metallic sphere electrode to regulate its surface charge. In the experiment, a high-voltage DC power module was used to apply an adjustable potential *U_s_* to an isolated metallic sphere with radius *R_s_*. Under this condition, the equivalent surface charge on the sphere is given by $Q_{s}=4\pi \varepsilon_{0}R_{s}U_{s}$, where *ε*_0_ is the vacuum permittivity.

**Figure 16:**
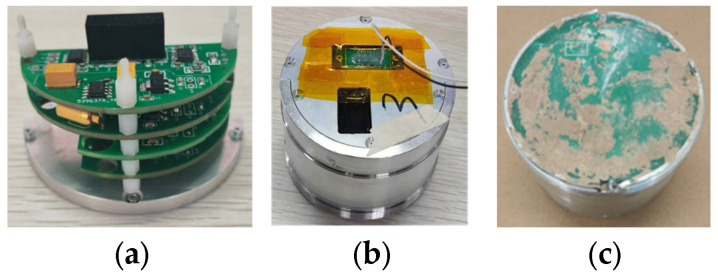
The electrostatic sensor: (**a**) assembly circuit; (**b**) after encapsulation; (**c**) after recovery.

The electrostatic sensor, as shown in Figure 16, had its internal circuitry encapsulated in epoxy resin and enclosed within a metallic housing to ensure structural integrity and provide effective electromagnetic shielding.

**Figure 17:**
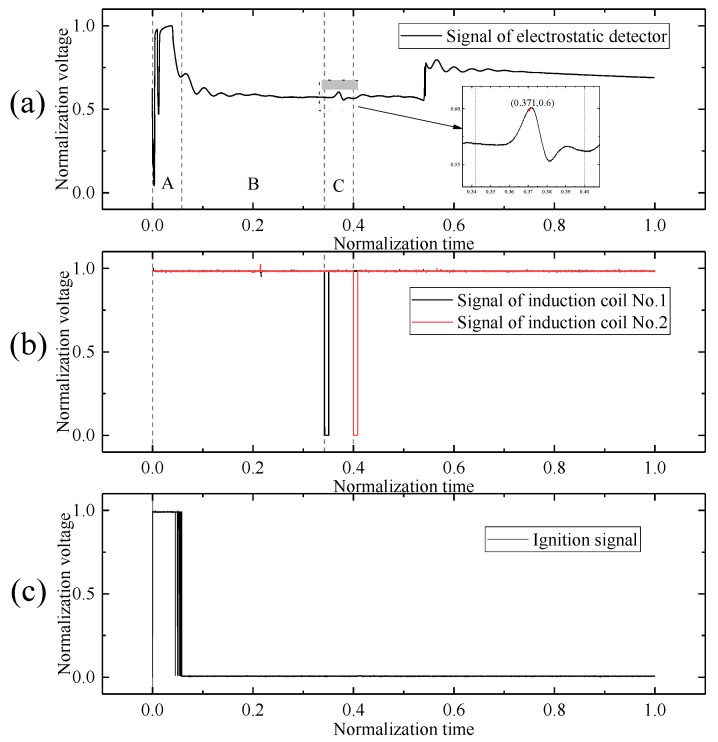
Signal outputs from different components during the full-flight measurement experiment: (**a**) electrostatic sensor signal; (**b**) induction coil signal; (**c**) ignition controller signal.

As shown in Figure 17, a representative set of experimental data was normalized for analysis. The in-flight data of the electrostatic sensor over the trajectory is presented in Figure 17a, and the flight is divided into three distinct phases: Phase A (powered phase), Phase B (coasting phase), and Phase C (encounter phase).

In Phase A, the electrostatic sensor’s output signal reaches saturation. This is attributed to the ignition of the rocket motor, which is triggered by the ignition controller. The combustion jet generates intense triboelectric charging and discharging on the rocket body. The induced coupling between the electrostatic sensor’s sensing electrode and the charged rocket body leads to signal saturation.

In Phase B, the propulsion ceases and the rocket transitions to free flight. The output signal stabilizes near the baseline. This occurs after motor burnout, where the charge exchange between the rocket and the surrounding environment is primarily due to frictional electrification between the rocket body and the air. As a result, the charging and discharging behavior becomes relatively steady.

In Phase C, the electrostatic sensor encounters the spherical metallic electrode target. The sensor responds to this interaction by producing a characteristic signal corresponding to the encounter event.

The encounter time was determined based on the output signal of the induction coils, as shown in Figure 17b. The pulse signals indicate that the rocket passed through the two induction coils at 0.342 and 0.400, respectively. Assuming negligible deceleration of the rocket between the two coils, the midpoint between these times, 0.371, is taken as the encounter time. This encounter moment occurs prior to the peak of the electrostatic sensor’s output signal.

The timing reference for both the electrostatic sensor and the ground-based data acquisition system was established by the ignition signal of the rocket motor, as shown in Figure 17c. The zero-time point in Figure 17 corresponds to the moment of engine ignition, which simultaneously marks the start of signal recording for the electrostatic sensor output.

**Figure 18:**
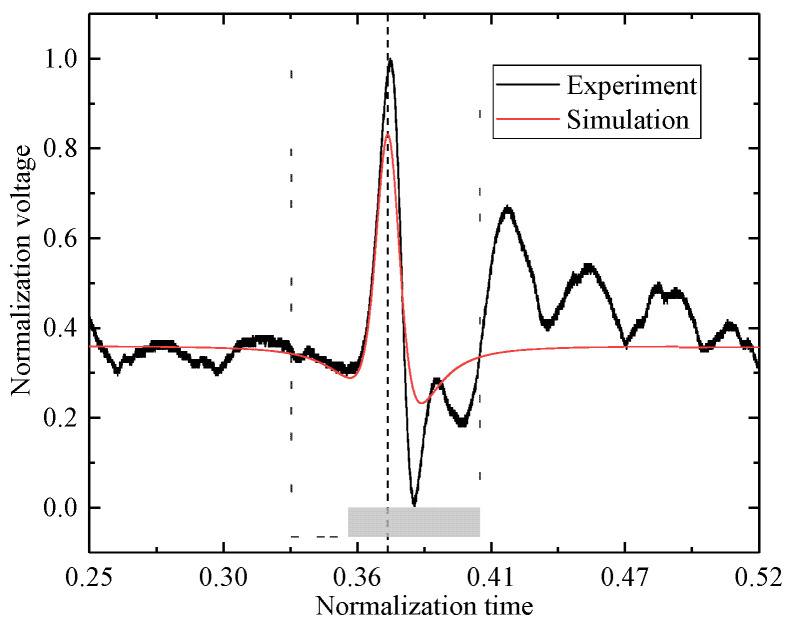
Comparison between experimental and simulated signals.

The simulation model was developed under the same parameter conditions as those used in the experiment, and the simulated output signal of the electrostatic sensor was compared with the experimental results, as shown in Figure 18. The theoretical and measured dynamic encounter times were used to align the simulated and experimental signals along the time axis. The encounter time is indicated by the vertical markers in Figure 18, and all signal results are presented in a normalized form.

Multiple sets of experiments were conducted using two types of electrostatic sensors, with their preamplifiers configured as either current amplifiers or charge amplifiers. The frequency response characteristics of the preamplifier circuits were measured following the procedure described in the study of [27]. During the experiments, the high-voltage DC power supply module maintained a constant output voltage. The encounter angle and distance were held constant, while the encounter velocity and timing were measured using an induction coil. The proposed modeling method was used to simulate the sensor output signals, and the simulation results were compared against experimental measurements in terms of correlation coefficients and peak-to-peak errors.

**Figure 19:**
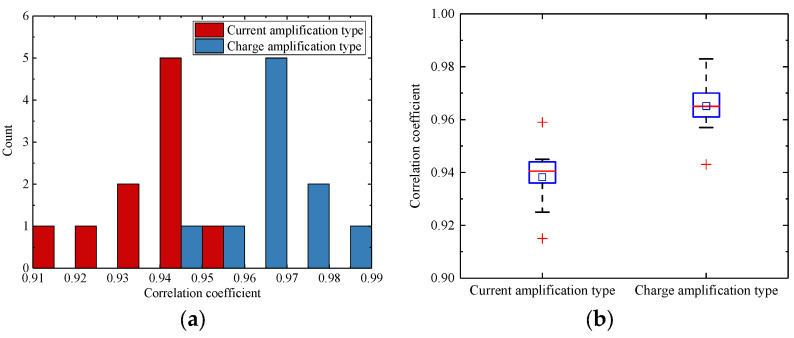
Distribution of the correlation coefficients: (**a**) histogram of the correlation coefficient distribution; (**b**) box plot of the correlation coefficient distribution.

The similarity between simulated and experimental signals was evaluated using the Pearson correlation coefficient *r.* A positive value of *r* indicates a positive correlation, while a negative value indicates a negative correlation. Typically, *r* > 0.8 indicates strong correlation, 0.5 ≤ *r* < 0.8 indicates moderate correlation, 0.3 ≤ *r* < 0.5 indicates weak correlation, and *r* < 0.3 suggests negligible correlation. Figure 19 shows the statistical distribution of the Pearson correlation coefficients between the experimental and simulated signals. The average Pearson correlation coefficient was 0.9517 with a standard deviation of 0.0177, and the minimum value observed was 0.915. The charge amplifier configuration exhibited a higher mean correlation coefficient (0.9651) compared to the current amplifier configuration (0.9382).

**Figure 20:**
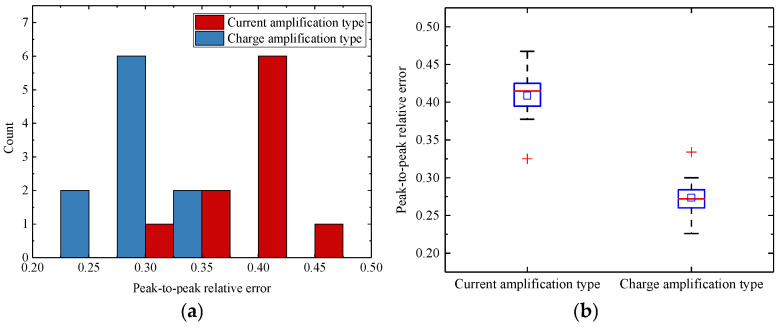
Distribution of the relative peak-to-peak errors: (**a**) histogram of the error distribution; (**b**) box plot of the error distribution.

Although the Pearson correlation coefficient effectively quantifies the similarity in signal trends, it is less sensitive to differences in signal amplitude. To assess amplitude discrepancies between simulated and experimental signals, the relative peak-to-peak error was calculated. Figure 20 illustrates the distribution of relative peak-to-peak errors. The overall mean error was 0.341 with a standard deviation of 0.0771, and the maximum observed value was 0.467. The charge amplifier configuration showed a significantly lower mean relative peak-to-peak error (0.2734) compared to the current amplifier configuration (0.4087).

Overall, the proposed model demonstrated good accuracy in estimating the key features of the electrostatic sensor output, with correlation coefficients exceeding 0.9, indicating strong consistency between simulation and experiment. However, there remained an amplitude discrepancy, with a mean peak-to-peak error of 34.1%.

## 4. Conclusions

This study proposes an analytical modeling method for electrically floating electrostatic sensor signals, calibrated under actual boundary conditions. The model incorporates the effects of encounter angle, miss distance, encounter velocity, and equivalent input resistance–capacitance parameters, enabling efficient prediction of sensor signals under multivariable coupling. A dynamic encounter model for the sensor output signal was developed, and dynamic encounter experiments were designed and conducted to obtain in-flight measurement data. Comparison of experimental and simulation results confirms the accuracy of the proposed model and demonstrates the feasibility of applying electrostatic sensing technology to low-altitude UAV detection and interception.

The main findings and conclusions are as follows:

1. The electrically floating electrostatic sensor with a flat-plate induction electrode exhibits both forward and backward spatial sensitivity. In the far-field region, its spatial sensitivity is symmetric with respect to the zero spatial sensitivity plane.
2. The simulated signal features show good agreement with experimental measurements, with correlation coefficients exceeding 0.9. This validates the accuracy of the proposed model and the effectiveness of the modeling methodology.
3. Experimental results demonstrate that the electrostatic sensor exhibits strong resistance to ground and sea clutter and can effectively detect low-altitude electrostatic targets during dynamic encounters. However, performance may be limited during powered phases due to electrostatic charging and discharging effects from rocket motor combustion and exhaust.

Further research will address the subproblems associated with dynamic encounter scenarios. A single electrostatic sensor can provide the timing information for the encounter event. However, due to its passive detection mechanism, it cannot independently determine the direction or range of the charged object. Future work will therefore focus on UAV detection using an array of electrostatic sensors. By integrating measurements through advanced multi-sensor signal fusion algorithms, we aim to develop methodologies capable of simultaneously estimating both the trajectory direction and the range of charged aerial targets. In addition, the investigation of interference mitigation strategies for electrically floating electrostatic sensors will be a key priority, with the objective of enhancing detection reliability under complex electromagnetic environments.

## Figures and Tables

**Table 1:** The size of the FEM model.

Diameter of Electrode	Diameter of the Model	Height of the Model
60 mm	100 mm	355 mm

**Figure A1:**
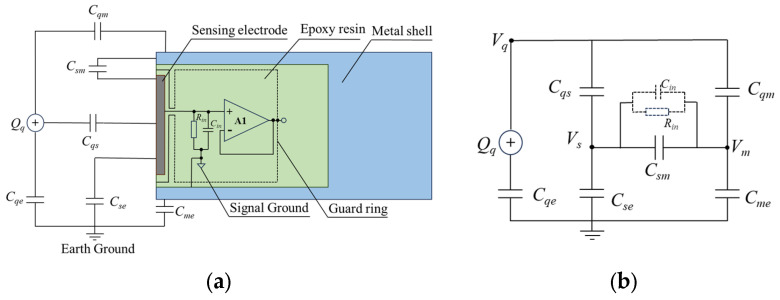
Capacitive coupling modeling of the floating-ground electrostatic sensor during charge measurement: (**a**) schematic diagram of capacitive coupling relationships; (**b**) equivalent mutual-capacitance circuit model.

**Figure A2:**
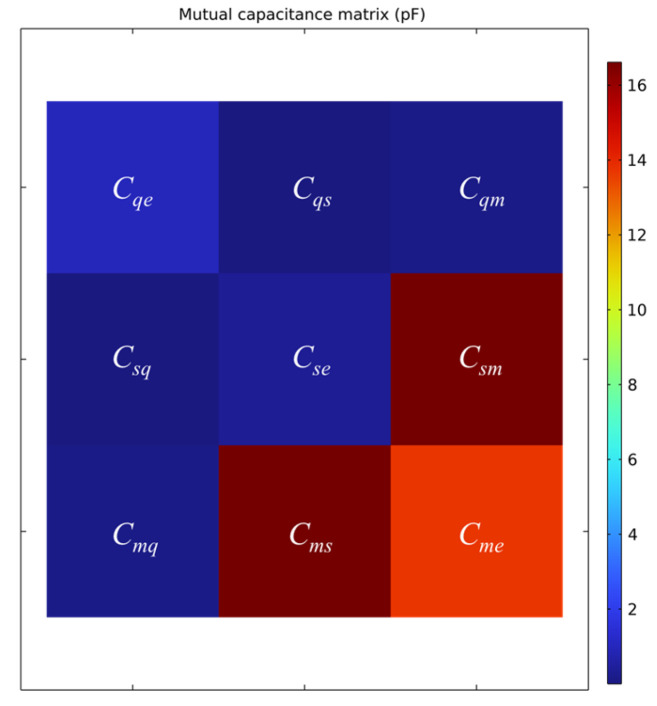
Calculated mutual capacitance matrix when the charged object is located at (1, 0, 0).

**Figure A3:**
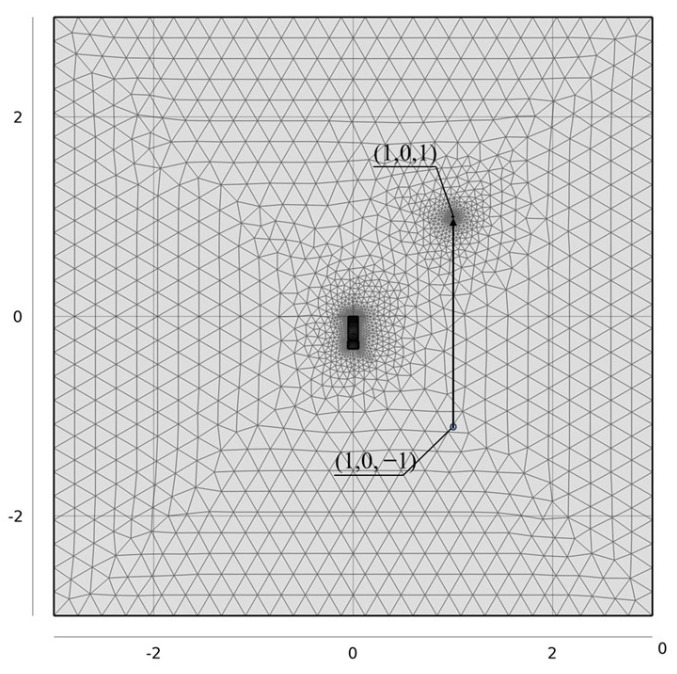
Schematic of the position variation in a charged object in the parameterized scanning simulation.

## Data Availability

The data are not publicly available due to the ongoing research.

## Appendix A

### Appendix A.1. Mutual Capacitance Model of a Floating Electrostatic Sensor

The electrostatic sensor described in this study operates in a floating-potential configuration during dynamic encounters. Its mutual capacitance matrix model differs from that of a grounded electrostatic sensor [28]. In this section, we establish the capacitive coupling model for the floating-potential sensor and derive the measurement model based on the mutual capacitance matrix.

As shown in Figure A1, The floating-potential sensor comprises a sensing electrode, conditioning circuit encapsulated in epoxy resin, and metallic housing. To increase the equivalent input impedance of the preamplifier, the sensor employs a bootstrapping technique [29,30]. This positive-feedback approach raises the effective input impedance and reduces stray capacitance *C_sm_*, between the sensing electrode and housing by surrounding the electrode and signal lines with guard electrodes and traces driven to the same potential as the input node. Active guarding enhances input impedance and minimizes leakage currents from electromagnetic interference.

The capacitances are defined as follows: *C_qe_*, *C_se_*, *C_me_* are the self-capacitances of the charged object, sensing electrode, and metallic housing to ground, respectively; *C_qs_* and *C_qm_* are the mutual capacitances between the charged object and the sensing electrode and between the charged object and the housing; and *C_sm_* is the mutual capacitance between the sensing electrode and the housing. In the equivalent circuit (Figure 19b), *V_q_*, *V_s_*, and *V_m_* represent the potentials of the charged object, sensing electrode, and metallic housing, respectively, with respect to the ground.

The Maxwell capacitance matrix defines and computes the capacitance coefficients for any two or more conductors [31], describing the relationship between the charge on any conductor and the potentials of all conductors in the system. The measurement system of a floating-potential electrostatic sensor interacting with a charged object can be expressed as follows:

(A1) $$\left(\begin{array}{c} Q_{q}\\ Q_{s}\\ Q_{m} \end{array}\right)=\left[\begin{array}{ccc} C_{qe}+C_{qs}+C_{qm} & -C_{qs} & -C_{qm}\\ -C_{sq} & C_{sq}+C_{se}+C_{sm} & -C_{sm}\\ -C_{mq} & -C_{ms} & C_{mq}+C_{ms}+C_{me} \end{array}\right]\left(\begin{array}{c} V_{q}\\ V_{s}\\ V_{m} \end{array}\right)$$

where *Q_q_*, *Q_s_*, and *Q_m_* are the total charges on the charged object, sensing electrode, and metallic housing, respectively. In the model, all three are assumed to be conductors.

### Appendix A.2. Finite Element Calculation of Induced Charge

From the equivalent circuit in Figure 19b, the induced charge *q*_1*sm*_ on the sensing electrode can be expressed as follows:

(A2) $$q_{1sm}=\left(V_{s}-V_{m}\right)C_{sm}$$

where *V_s_* and *V_m_* are the simulated potentials of the sensing electrode and housing, and *C_sm_* is the mutual capacitance between them. These quantities are obtained through finite element simulations and substituted into Equation (A2) to compute the numerical value of *q*_1*sm*_. The simulation parameters are listed in Table A1.

**Table A1:** Simulation parameter settings.

*Q_q_*	*Q_s_*	*Q_m_*	Relative Permittivity of Air	Relative Permittivity of Epoxy Resin
1 C	0 C	0 C	1	4.5

In the numerical example, a trajectory between points (1, 0, −1) and (1, 0, 1) on the XOZ-plane is selected for parametric scanning. The step size is 0.1. At each position, *V_s_*, *V_m_*, and *C_sm_* are computed. When the charged object is located at (1, 0, 0), the corresponding mutual capacitance matrix is shown in Figure A2. The motion path of the charged object during the parametric scan is illustrated in Figure A3.

The spatial sensitivity of the electrostatic sensor is defined as the absolute value of the induced charge generated on the sensing electrode by a unit external point charge. In the finite element simulations, when the charge *Q_q_* of the moving object is set to 1 C, *S_FEM_* is numerically equal to |*q*_1*sm*_|. The finite element results for *S_FEM_* at various positions along the path from (1, 0, −1) to (1, 0, 1) are listed in Table A2.

**Table A2:** Finite element computation results for a moving charged object at different positions.

Coordinate of Charged Object	*V_s_* (V)	*V_m_* (V)	*C_sm_* (pF)	*q*_1*sm*_ (C)	*S_f_*
(0, 1, −1)	5.77092 × 10^9^	5.80299 × 10^9^	16.58532	−5.31815 × 10^−4^	5.31815 × 10^−4^
(0, 1, −0.8)	6.40789 × 10^9^	6.43997 × 10^9^	16.58349	−5.32037 × 10^−4^	5.32037 × 10^−4^
(0, 1, −0.6)	6.93841 × 10^9^	6.96638 × 10^9^	16.5841	−4.63807 × 10^−4^	4.63807 × 10^−4^
(0, 1, −0.4)	7.25821 × 10^9^	7.27655 × 10^9^	16.58082	−3.03972 × 10^−4^	3.03972 × 10^−4^
(0, 1, −0.2)	7.29716 × 10^9^	7.30117 × 10^9^	16.58233	−6.64799 × 10^−4^	6.64799 × 10^−4^
(0, 1, 0)	7.04328 × 10^9^	7.03163 × 10^9^	16.57827	1.93117 × 10^−4^	1.93117 × 10^−4^
(0, 1, 0.2)	6.55901 × 10^9^	6.53481 × 10^9^	16.58197	4.01259 × 10^−4^	4.01259 × 10^−4^
(0, 1, 0.4)	5.94301 × 10^9^	5.91191 × 10^9^	16.58249	5.15732 × 10^−4^	5.15732 × 10^−4^
(0, 1, 0.6)	5.28939 × 10^9^	5.25652 × 10^9^	16.584	5.45085 × 10^−4^	5.45085 × 10^−4^
(0, 1, 0.8)	4.66207 × 10^9^	4.63079 × 10^9^	16.58452	5.18783 × 10^−4^	5.18783 × 10^−4^
(0, 1, 1)	4.09228 × 10^9^	4.0641 × 10^9^	16.58538	4.6736 × 10^−4^	4.6736 × 10^−4^
